# Supplementary material for: Identifying the immune interactions underlying HLA class I disease associations
Source: eLife. 2020 Apr 2;9:e54558. doi: 10.7554/eLife.54558 (PMC7253178; doi:10.7554/eLife.54558)
Supplement: Supplementary file 1. [file elife-54558-supp1.docx]

## Worked examples of the Fraction Shared Calculations.

### Worked example 1.

Imagine we are interested in whether the protective effect of HLA-A*02:07 in the HTLV-1 –infected Kagoshima cohort is attributed to TCR, iKIR, aKIR, LILRB1 or LILRB2 binding.

First we predict the HTLV-1 peptides that bind A*02:07, for concreteness consider 9mers. There are 161 9-mers predicted to bind A*02:07:

| Peptide | Source Protein | Peptide | Source Protein | Peptide | Source Protein | Peptide | Source Protein |
| --- | --- | --- | --- | --- | --- | --- | --- |
| RICPINYSL | Gag | LLLLSEATM | Pol | LLFLPFQIL | Rof | LTMLIISPL | Tof |
| RVNEILHIL | Gag | SLISHGLPV | Pol | QILSGLLFL | Rof | LIISPLPRV | Tof |
| ILIQTQAQI | Gag | LTYDAVPTV | Pol | ILSGLLFLL | Rof | RLVPHLWGT | Tof |
| YVEPTAPQV | Gag | ALPELQALL | Pol | LLFLLFLPL | Rof | LIISPLPRV | P13 |
| WQMKDLQAI | Gag | ALLGEIQWV | Pol | FLLFLPLFF | Rof | RLVPHLWGT | P13 |
| FMQTIRLAV | Gag | RLVQTLPLL | Pol | LFLPLFFSL | Rof | SLGDYVRPA | Rex |
| LLQYLCSSL | Gag | LLGAIMLTL | Pol | FLPLFFSLP | Rof | IVTPYWPPV | Rex |
| LQYLCSSLV | Gag | FQSKEQWPL | Pol | PLFFSLPLL | Rof | SMDALSAQL | Rex |
| QLWLAAFAA | Gag | QLLASAVLL | Pol | LFFSLPLLL | Rof | EMDTWNPPL | Rex |
| FVERLNIAL | Gag | LLASAVLLL | Pol | LLLSPSLPI | Rof | EMDTWNPPL | P21 |
| LLLDLPADI | Gag | VLLLDKYTL | Pol | MLFRLLSPL | P12 | QSLLFGYPV | Tax |
| FLATLILFF | Env | YTLQSYGLL | Pol | RLLSPLSPL | P12 | LLFGYPVYV | Tax |
| ILFFQFCPL | Env | KTAAPLAPV | Pol | LLSPLSPLA | P12 | VIGSALQFL | Tax |
| ALQPPCPNL | Env | KALMPVFTL | Pol | ALTALLLFL | P12 | ALQFLIPRL | Tax |
| SLYLFPHWT | Env | ALMPVFTLS | Pol | LTALLLFLL | P12 | FLIPRLPSF | Tax |
| YLGCQSWTC | Env | ILWDKQILS | Pol | LLLFLLPPS | P12 | TLGQHLPTL | Tax |
| FLNTEPSQL | Env | ILSQRSFPL | Pol | LLLFLPFQI | P12 | TLSFPDPGL | Tax |
| QLPPTAPPL | Env | ELLGLLHGL | Pol | LLFLPFQIL | P12 | YTLWGGSVV | Tax |
| LLTLVQLTL | Env | YLYHYLRTL | Pol | QILSGLLFL | P12 | VVCMYLYQL | Tax |
| VLYSPNVSV | Env | SQAPFQALL | Pol | ILSGLLFLL | P12 | YLYQLSPPI | Tax |
| SLALPAPHL | Env | LLSRKVVYL | Pol | LLFLLFLPL | P12 | LSPPITWPL | Tax |
| ALPAPHLTL | Env | NLPDPISRL | Pol | FLLFLPLFF | P12 | ITWPLLPHV | Tax |
| SLILPPFSL | Env | RLNALTDAL | Pol | LFLPLFFSL | P12 | QLGAFLTNV | Tax |
| FSLSPVPTL | Env | LLITPVLQL | Pol | FLPLFFSLP | P12 | ILPEDCLPT | Tax |
| AVPVAVWLV | Env | VLQLSPAEL | Pol | PLFFSLPLL | P12 | GLLPFHSTL | Tax |
| LLFWEQGGL | Env | TLYRLHVWV | Pol | LFFSLPLLL | P12 | KAYHPSFLL | Tax |
| ALQTGITLV | Env | SLLQAIAHL | Pol | LLLSPSLPI | P12 | IQYSSFHSL | Tax |
| ITLVALLLL | Env | FLNMCTSLA | Pol | LLLFLPFQI | p8 | LLFEEYTNI | Tax |
| TLVALLLLV | Env | ALSIALWTI | Pol | LLFLPFQIL | p8 | ELVDGLLSL | HBZ_SP1 |
| ALFSSNTPL | Pro | WQLHHSPRL | Pol | QILSGLLFL | p8 | GLLSLEEEL | HBZ_SP1 |
| KLTSLPVLI | Pro | HLAFQLSSI | Rof | ILSGLLFLL | p8 | AVLDGLLSL | HBZ_SP1 |
| RLPFRTTPI | Pro | QLLHFFFPS | Rof | LLFLLFLPL | p8 | MVNFVSVGL | HBZ_US |
| ILPIQAPAV | Pro | LLHFFFPST | Rof | FLLFLPLFF | p8 | ELVDGLLSL | HBZ_US |
| IQAPAVLGL | Pro | FFFPSTMLF | Rof | LFLPLFFSL | p8 | GLLSLEEEL | HBZ_US |
| RLQALQHLV | Pol | MLFRLLSPL | Rof | FLPLFFSLP | p8 | AVLDGLLSL | HBZ_US |
| YTGPGNNPV | Pol | RLLSPLSPL | Rof | PLFFSLPLL | p8 | ELVDGLLSL | HBZ_SP2 |
| SLPTTLAHL | Pol | LLSPLSPLA | Rof | LFFSLPLLL | p8 | GLLSLEEEL | HBZ_SP2 |
| FQPYFAFTV | Pol | ALTALLLFL | Rof | LLLSPSLPI | p8 | AVLDGLLSL | HBZ_SP2 |
| QLAHILQPI | Pol | LTALLLFLL | Rof | ALCCFAFSA | Tof |  |  |
| ILQYMDDIL | Pol | LLLFLLPPS | Rof | FLLATSAAF | Tof |  |  |
| YMDDILLAS | Pol | LLLFLPFQI | Rof | FLFKYSAVC | Tof |  |  |

There are thus 161 TCR recognition motifs:

| ICPINL | LTLVQL | LPTTLL | ALMPVL | LLHFFS | FFSLPL | LLSPSI | IISPLV | TWPLLV |
| --- | --- | --- | --- | --- | --- | --- | --- | --- |
| VNEILL | LYSPNV | QPYFAV | LMPVFS | LHFFFT | LLSPSI | LLFLPI | LVPHLT | LGAFLV |
| LIQTQI | LALPAL | LAHILI | LWDKQS | FFPSTF | LFRLLL | LFLPFL | LGDYVA | LPEDCT |
| VEPTAV | LPAPHL | LQYMDL | LSQRSL | LFRLLL | LLSPLL | ILSGLL | VTPYWV | LLPFHL |
| QMKDLI | LILPPL | MDDILS | LLGLLL | LLSPLL | LSPLSA | LSGLLL | MDALSL | AYHPSL |
| MQTIRV | SLSPVL | LLLSEM | LYHYLL | LSPLSA | LTALLL | LFLLFL | MDTWNL | QYSSFL |
| LQYLCL | VPVAVV | LISHGV | QAPFQL | LTALLL | TALLLL | LLFLPF | MDTWNL | LFEEYI |
| QYLCSV | LFWEQL | TYDAVV | LSRKVL | TALLLL | LLFLLS | FLPLFL | SLLFGV | LVDGLL |
| LWLAAA | LQTGIV | LPELQL | LPDPIL | LLFLLS | LLFLPI | LPLFFP | LFGYPV | LLSLEL |
| VERLNL | TLVALL | LLGEIV | LNALTL | LLFLPI | LFLPFL | LFFSLL | IGSALL | VLDGLL |
| LLDLPI | LVALLV | LVQTLL | LITPVL | LFLPFL | ILSGLL | FFSLPL | LQFLIL | VNFVSL |
| LATLIF | LFSSNL | LGAIML | LQLSPL | ILSGLL | LSGLLL | LLSPSI | LIPRLF | LVDGLL |
| LFFQFL | LTSLPI | QSKEQL | LYRLHV | LSGLLL | LFLLFL | LCCFAA | LGQHLL | LLSLEL |
| LQPPCL | LPFRTI | LLASAL | LLQAIL | LFLLFL | LLFLPF | LLATSF | LSFPDL | VLDGLL |
| LYLFPT | LPIQAV | LASAVL | LNMCTA | LLFLPF | FLPLFL | LFKYSC | TLWGGV | LVDGLL |
| LGCQSC | QAPAVL | LLLDKL | LSIALI | FLPLFL | LPLFFP | TMLIIL | VCMYLL | LLSLEL |
| LNTEPL | LQALQV | TLQSYL | QLHHSL | LPLFFP | LFFSLL | IISPLV | LYQLSI | VLDGLL |
| LPPTAL | TGPGNV | TAAPLV | LAFQLI | LFFSLL | FFSLPL | LVPHLT | SPPITL |  |

And 161 KIR recognition motifs:

| IYSL | LLTL | LAHL | AFTL | LFPS | FLLL | LLPI | IPRV | TPHV |
| --- | --- | --- | --- | --- | --- | --- | --- | --- |
| VHIL | LVSV | QFTV | LTLS | LPST | LLPI | LFQI | LWGT | LTNV |
| LAQI | LPHL | LQPI | LILS | FMLF | LSPL | LQIL | LRPA | LLPT |
| VPQV | LLTL | LDIL | LFPL | LSPL | LSPL | ILFL | VPPV | LSTL |
| QQAI | LFSL | MLAS | LHGL | LSPL | LPLA | LFLL | MAQL | AFLL |
| MLAV | SPTL | LATM | LRTL | LPLA | LLFL | LLPL | MPPL | QHSL |
| LSSL | VWLV | LLPV | QALL | LLFL | TFLL | LLFF | MPPL | LTNI |
| QSLV | LGGL | TPTV | LVYL | TFLL | LPPS | FFSL | SYPV | LLSL |
| LFAA | LTLV | LALL | LSRL | LPPS | LFQI | LSLP | LVYV | LEEL |
| VIAL | TLLL | LQWV | LDAL | LFQI | LQIL | LPLL | IQFL | VLSL |
| LADI | LLLV | LPLL | LLQL | LQIL | ILFL | FLLL | LPRL | VVGL |
| LLFF | LTPL | LLTL | LAEL | ILFL | LFLL | LLPI | LPSF | LLSL |
| LCPL | LVLI | QWPL | LVWV | LFLL | LLPL | LFSA | LPTL | LEEL |
| LPNL | LTPI | LVLL | LAHL | LLPL | LLFF | LAAF | LPGL | VLSL |
| LHWT | LPAV | LLLL | LSLA | LLFF | FFSL | LAVC | TSVV | LLSL |
| LWTC | QLGL | LYTL | LWTI | FFSL | LSLP | TSPL | VYQL | LEEL |
| LSQL | LHLV | TGLL | QPRL | LSLP | LPLL | IPRV | LPPI | VLSL |
| LPPL | TNPV | TAPV | LSSI | LPLL | FLLL | LWGT | SWPL |  |

We then go through every individual in the Kagoshima cohort and calculate their nearness to A*02:07 by the 5 metrics.

Consider an individual with the following KIR and HLA genotype:

| ID | A1 | A2 | B1 | B2 | C1 | C2 | KIR2DL1 | KIR2DL3 | KIR3DL1 | KIR3DL2 | KIR2DL2 | KIR2DS2 | KIR2DS3 | KIR2DS4 | KIR3DS1 | KIR2DL4 |
| --- | --- | --- | --- | --- | --- | --- | --- | --- | --- | --- | --- | --- | --- | --- | --- | --- |
| 1 | A1101 | A2402 | B0702 | B0702 | C0702 | C0702 | 1 | 1 | 1 | 1 | 0 | 0 | 1 | 1 | 0 | 1 |

First we calculate their nearness by the TCR measure. We take each HLA allele in turn and calculate its TCR.FS. The first HLA molecule, A*11:01, binds 100 peptides there are thus 100 TCR binding motifs:

| SYDFHK | NINLHK | TSLPVR | ISPNHY | QGDITK | FFSARR | IPSLRR | MRKYSR | AADVAK |
| --- | --- | --- | --- | --- | --- | --- | --- | --- |
| IALETR | LEPSIK | TSCLVK | SLYCAR | GAISAK | FSSSFK | VWRLCR | SVVCMY | QIAEYR |
| LLASLK | LSTWHY | VLYLPK | TTVVFK | ISATQK | SSSFLY | LIISPR | AFLTNY | IAEYLK |
| TQAQIR | SSSTPY | TWSGRR | SAVLLK | QAIAHK | SFSRSR | VWTESR | FLTNVK | AADVAK |
| AALPGK | SMSLAK | ITQYSK | LHHSHK | ILKTLK | SFFRFS | SFRIPR | SSSFIK |  |
| LAYSNK | SLLHEK | TQYSQR | AAPLAK | LYKYFK | AFSPGR | IPSLRR | IFHKFK |  |
| ACQTWK | QLTQAK | ALQHLK | AAYILK | QTHWYK | LSSSKK | VWRLCR | ISLLFK |  |
| VLVVQK | QAIVKK | NNPVFK | LNIFLK | SAQWIR | LSFNSK | TCLETK | QIAEYR |  |
| GVSSYK | QYAAQR | SLPTTH | LLPRLR | WIPWRK | SSGHDK | TWDPIR | IAEYLK |  |
| LFPHWK | LAGPCR | FFQIPK | LPRLLK | TMLFRS | LIISPR | ALQFLR | AADVAK |  |
| TGAVSY | QLRHLR | TMASLH | VVYLHR | SAPCLR | VWTESR | LPSFPR | QIAEYR |  |
| AVSSPK | FSSNTK | QQTPGK | TEASNR | SAAFFR | SFRIPR | SFLQAK | IAEYLK |  |

None of the A*11:01 TCR motifs are in the A*02:07 TCR motifs list. Thus

$$TCR.FS\left(index=A0207,query=A1101,proteome=HTLV\right)=0$$

We repeat this for all HLA alleles carried by the individual:

$$\begin{matrix} TCR.FS\left(index=A0207,query=A2402,proteome=HTLV\right)=\frac{14}{161} \\ =0.087 \\ TCR.FS\left(index=A0207,query=B0702,proteome=HTLV\right)=\frac{9}{161} \\ =0.056 \\ TCR.FS\left(index=A0207,query=C0702,proteome=HTLV\right)=\frac{44}{161} \\ =0.273 \\ \end{matrix}$$

The allele closest to A*02:07 in TCR binding space with respect to the HTLV-1 proteome is thus C*07:02 and so this individual is given a TCR.FS=0.273.

Next, we calculate their nearness by the NK metrics. The index allele, A*02:07, is not thought to bind any inhibitory KIR. Working through each of the individual’s HLA molecules: A*24:02 contains a Bw4-80I motif and is thought to bind the iKIR KIR3DL1, A*11:01 is thought to bind KIR3DL2, B07:02 is not thought to bind any iKIR and C07:02 (which contains the C1 motif) is thought to bind KIR2DL2/L3. B07:02 is thus the allele closest to A*02:07 in iKIR binding space. Since neither molecule binds any iKIR the KIR binding motif is irrelevant and both alleles are considered identical (in terms of iKIR binding) and the iKIR.FS=1. Similarly, for activating KIR, A*02:07 is not thought to bind any activating KIR, neither is B*07:02 and so B*0702 is the nearest molecule this person possesses to A*0207 in aKIR binding space and (by the same reasoning as for iKIR) aKIR.FS=1.

Finally, we calculate the LILRB1 and LILRB2 binding scores. This is done on a locus by locus basis so, in this example as the index is A*02:07, we consider the similarity of the two HLA-A molecules possessed by this individual, i.e. A*11:01 and A*2402. From Table S1 in the Supplementary Information to Bashirova et al we find that the binding of LILRB1 to A*02:07 is not measured and so we calculate the similarity score for all A*02 molecules and take the mean. The binding of LILRB1 to A*11:01 is 0.48 and A*24:02 is 0.43; giving us the following:

| Index allele | Query allele | Index LILRB1 binding score | Query LILRB1 binding score | Calculation | Similarity score |
| --- | --- | --- | --- | --- | --- |
| A*02:01 | A*11:01 | 0.11 | 0.48 | $\frac{\left\vert0.48-0.11\right\vert}{\left\vert0.63-0.03\right\vert}$ | 0.383 |
| A*02:03 | A*11:01 | 0.17 | 0.48 | $\frac{\left\vert0.48-0.17\right\vert}{\left\vert0.63-0.03\right\vert}$ | 0.483 |
| A*02:06 | A*11:01 | 0.28 | 0.48 | etc | 0.667 |
| A*02:01 | A*24:02 | 0.11 | 0.43 |  | 0.467 |
| A*02:03 | A*24:03 | 0.17 | 0.43 |  | 0.567 |
| A*02:06 | A*24:04 | 0.28 | 0.43 |  | 0.75 |

And so, the LILRB1 similarity score for A*0207 to A*1101 is 0.511, and the LILRB1 similarity score for A*0207 to A*2402 is 0.594. This individual thus gets a LILRB1 similarity score of 0.594. Repeating this calculation for LILRB2 we find that this individuals LILRB2 binding score is 0.906. This example is represented schematically in Figure 1.

### Worked example 2.

As a second worked example we consider the similarity metric for the same individual but to the index allele HLA-C*08:01.

There are 301 9-mers predicted to bind C*08:01 and thus 301 TCR recognition motifs. As before we calculate the TCR.FS for each allele carried by individual 1:

$$\begin{matrix} TCR.FS\left(index=C0801, query=A1101,proteome=HTLV\right)=0 \\ TCR.FS\left(index=C0801,query=A2402,proteome=HTLV\right)=\frac{34}{301} \\ =0.113 \\ TCR.FS\left(index=C0801,query=B0702,proteome=HTLV\right)=\frac{63}{301} \\ =0.209 \\ TCR.FS\left(index=C0801,query=C0702,proteome=HTLV\right)=\frac{104}{301} \\ =0.346 \\ \end{matrix}$$

The allele closest to C*08:01 in TCR binding space with respect to the HTLV-1 proteome is thus C*07:02 and so this individual is given a TCR.FS=0.346.

Next, we calculate their nearness by the NK metrics. The index allele, C*08:01, contains the C1 binding motif and binds the inhibitory KIR KIR2DL2/L3. The individual considered also has an allele with a C1 binding motif (C*07:01) as well as a ligating KIR (KIR2DL3). C*0801 has 301 KIR recognition motifs, C*0702 has 263 of which 112 are C*0801 motifs. The iKIR.FS allocated is thus

iKIR.FS (index=C0801, query=C0701, proteome=HTLV) = 112/301 = 0.372

Moving on to the aKIR.FS, C*0801 and C*0702 both bind KIR2DS2. However, this individual is *KIR2DS2*-negative and so

aKIR.FS (index=C0801, query=C0701, proteome=HTLV) = 0.

Finally, we calculate the LILRB1 and LILRB2 binding scores. The index is C*0801, so we consider the similarity of the HLA-C molecules possessed by this individual, i.e. C*0701. The LILRB1 binding scores to C*0801 and C*0702 are 0.22 and 0.15. The LILRB1 similarity score is thus

LILRB1.S (index=C0801, query=C0702) = 1- |0.22-0.15|/[0.63-0.03| = 0.883

Similarly, the LILRB2 similarity score is

LILRB2.S (index=C0801, query=C0702) = 1- |0.36-0.62|/[0.98-0.09| = 0.708.
